# Circadian Pattern of Melatonin MT1 and MT2 Receptor Localization in the Rat Suprachiasmatic Nucleus

**DOI:** 10.5334/jcr.ab

**Published:** 2015-03-10

**Authors:** Nermien E. Waly, Richard Hallworth

**Affiliations:** Department of Physiology, School of Medicine, Assiut University, Egypt; Department of Biomedical Sciences, Creighton University, United States

**Keywords:** circadian rhythm, melatonin, MT1, MT2, SCN, melatonin receptors, immunohistochemistry

## Abstract

The suprachiasmatic nucleus (SCN) is the master circadian pacemaker. The pineal hormone melatonin is involved in the regulation of circadian phase. As a part of the circadian system, its synthesis and secretion is under SCN control. On the other hand, melatonin feeds back on the SCN to regulate its function. Melatonin has two specific windows of time at which it regulates SCN function, namely dusk and dawn. It has been suggested that melatonin exerts its effect on the SCN during that specific window of time via one or both of its specific receptors, MT1 or MT2. The hypothesis that the density of these receptors varies across the circadian cycle was tested. Using immunohistochemistry with receptor-specific antibodies, the localization and distribution of melatonin receptors MT1 and MT2 was studied in the SCN at different Zeitgeber times (ZT): ZT 11–13 (dusk), 23–01 (dawn), 5–7 (mid-day), and 17–19 (midnight). Our results show that MT1 receptor density significantly increased at dusk relative to dawn and midnight (p<0.01 and p<0.001 respectively). Although MT1 receptors were widespread in the SCN and parts of the optic chiasm at dusk, they were restricted to the SCN during the mid-day period. MT2 receptors were not detected in the SCN. Thus, we find that melatonin receptor MT1 density and distribution varies with circadian time. This creates a time window during which melatonin can affect the operation of the SCN. We also find that melatonin regulates SCN function via MT1 receptors with a minimal role for MT2.

## 1. Introduction

Melatonin, “the chemical expression of darkness” [20], is a neuroendocrine hormone synthesized and secreted by the pineal gland [4,25]. There is a circadian rhythm in melatonin synthesis and secretion that peaks at night and troughs during the day [20]. The circadian rhythm of melatonin synthesis is driven by the intrinsic circadian activity of the suprachiasmatic nucleus (SCN). This small paired nucleus in the anterior hypothalamus is entrained to a 24-hour period by the light/dark cycle (LD) [14]. The SCN is strategically positioned to receive visual input for LD entrainment through the retino-hypothalamic tracts [22]. On the other hand, melatonin feeds back on the SCN to regulate its function by modulation of SCN electrical activity [11,12], resetting the clock [5,6] and synchronizing locomotor activity [17,19,24]. Melatonin can reset the circadian clock only at temporally distinct times, dusk and dawn. When melatonin is administered at dusk, it phase advances the firing rate of SCN neurons in rat and mouse [5,9]. McArthur et al. also described a behavioral activity phase advance in response to melatonin between circadian time (CT) 23 and 01 (dawn) [12].

Melatonin is believed to exert these effects via two specific, high affinity, guanine nucleotide binding protein (G protein) coupled receptors (GPCR) in the SCN, MT1 and MT2 [21]. MT1 mRNA is expressed in the SCN, however the expression of MT2 receptor mRNA has been more difficult to evaluate [15,18,23,26]. There is evidence that there is a circadian rhythm of the expression of the MT1 receptor mRNA as well as of melatonin binding in the rat brain. Melatonin receptor MT1 mRNA expression exhibited a distinct circadian rhythm peaking during subjective night [10]. However, another study found that MT1 mRNA peaked 3 hours ahead of melatonin binding [10,15]. This result however has been controversial, as in another study there was no statistically significant variation in mRNA levels across the circadian cycle [26]. Poirel et al. were not able to detect any expression of MT2 receptors mRNA in SCN [18]. Using binding studies, MT2 receptors in the SCN are expressed in very low levels [3,9].

The above-mentioned studies used methods such as RT-PCR or in situ hybridization (ISH) as well as receptor binding to infer the presence of both MT1 and MT2 receptors in the SCN tissue. A circadian rhythm of receptor protein synthesis and localization has not been demonstrated due to the lack of specific antibodies for MT1 and MT2 receptors. Such a rhythm would explain the temporal gating of melatonin efficacy in modulating behavioral rhythm and electrophysiology. Therefore, this study describes the use of immunohistochemistry in the study of the circadian distribution of melatonin receptors MT1 and MT2 in the SCN.

## 2. Experimental procedure

### 2.1. Animals and tissue preparation

Long Evans rats (Charles River, Wilmington, MD), 4–6 weeks old, were entrained for at least 2 weeks to a 12/12 LD cycle prior to the experiments. Animals were housed in groups of 4 rats per cage and provided with rodent chow and water ad libitum. Animal care and handling was performed in conformance with approved protocols of the Creighton University School of Medicine Institutional Animal Care and Use Committee.

For immunoblotting, 4 rats were decapitated rapidly after 1 minute of exposure to CO_2_ at dusk (ZT 11–13). The brains were quickly removed and cut into blocks containing the hypothalamus. Blocks were chilled on ice-cold artificial cerebrospinal fluid (a-CSF). One 400–500 mm thick coronal slice containing the SCN was prepared from each brain using a vibrating tissue slicer (TCI, St Louis, MO). The slices were further reduced into punches that contain SCN tissue under a dissecting microscope (Olympus SZX12, Japan). The reduced hypothalamic slices were then homogenized using lysis buffer (10 mM Tris pH 7.4, 150 mM sodium chloride, 10% glycerol, 1% Triton X-100, 1 mM EDTA, Complete Protease Inhibitor tablets [Roche, Mannheim, Germany], 1 mM sodium orthovanadate, 1.5 mM EGTA and 10 mM sodium fluoride) for 20 minutes, then were centrifuged for 10 minutes at 14000 round per minute (rpm). The supernatant was then removed and stored at – 20ºC until used.

For immunohistochemistry as well as immunofluorescence, rats were anesthetized at the desired circadian time (CT) with Nembutal Sodium Solution (100 mg/kg) (Abbott Laboratories, Chicago, IL, USA), then fixed by cardiac perfusion using heparinized saline followed by 4% paraformaldehyde fixative. Brains were removed and kept in the fixative solution for at least one hour. Brains were then rinsed several times with PBS and kept at 4°C until used.

### 2.2. Immunohistochemistry (IHC)

IHC was performed on free floating sections (70 µM) using standard avidin–biotin complex (ABC) methods as previously described [1]. Coronal brain sections were prepared using a vibratome (TCI, St Louis, MO) and incubated at 4°C in PBS in 24-well plates. Sections were washed several times with 0.3% Triton X-100 in phosphate buffer saline (PBS-T) before treatment with 0.3% H_2_O_2_/PBS for 30 minutes. Non-specific binding sites were blocked using 2% normal goat serum in PBS for 1 hour. After thorough rinsing with PBS-T for at least 15 minutes, sections were incubated for about 36 hours at 4ºC with MT1 or MT2 rabbit anti-human polyclonal antibody serum in PBS (Lifespan Biosciences Inc., WA) at 1:500 concentrations and washed 3 times with PBS-T for at least 10 minutes. Sections were then incubated with biotinylated goat anti-rabbit IgG (1:200; Vector Laboratories, Burlingame, CA) for 1 hour. After washing for 10 minutes with PBS-T, sections were incubated for 2 hours in ABC solution (1:100; Vector Laboratories, Burlingame, CA). The reaction product was visualized by incubating the sections with 3, 3’ diaminobenzidine (DAB) (Vector Laboratories, Burlingame, CA) for 4 to 5 minutes in PBS. Sections were then mounted on slides and covered with mounting media (5 ml PBS, 5 ml glycerol, 0.1 g n-propylgalate) then covered with cover slips before visualization. Specimens were photographed using an Axioskop II microscope (Carl Zeiss Jena, Jena, Germany) equipped with 40x and 100x objectives. Negative controls were performed by omission of the primary antibody. Positive controls are described in the results. Images were obtained using a Spot RT digital camera (Diagnostic Instruments, Sterling Heights, MI). Images were prepared using Adobe Photoshop (Adobe Systems®, San Jose, CA).

### 2.3. Immunoblotting

Immunoblots were performed under standard conditions with the same antibodies used for immunohistochemistry. A uniform amount of homogenized hypothalamus (25 µg) was loaded onto each lane of a 10% polyacrylamide gel (Biorad, Hercules, CA). The protein content was determined using a microplate reader and protein (BSA) standards (MPM, microplate reader, Biorad). The lanes were run at 100 V and 250 mA for 60 min. in sodium dodecyl sulphate (SDS) electrophoresis running buffer. Proteins were then transferred from the gels to nitrocellulose sheets at 100 V and 100 mA for 60 min. in a transfer buffer containing Trizma base and glycine (Sigma, St. Louis, MO). The nitrocellulose sheets containing the transferred protein lanes were then cut into strips and were exposed to one of the primary antibodies overnight at 4°C. After thorough rinsing with 1% milk in PBS, the secondary antibody (anti-rabbit IgG linked to biotin, Cell Signaling Technology, Beverly, MA), was added and the strips were incubated on a rocker for 1 h at room temperature. The protein strips were then rinsed again three times in 1% milk in PBS for 10 min. and twice in PBS for 10 min. They were then treated with SuperSignal West Pico Chemiluminiscent Substrate (Pierce Biotechnology, Inc., Rockford, IL) for 5 min. and exposed to a CL-XPosure blue x-ray film (Pierce Biotechnology) for 30 s.

**Fig. 1:**
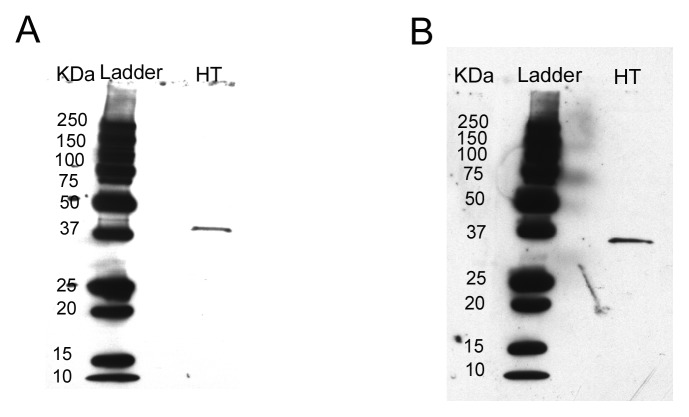
Immunoblotting of rat hypothalamus (HT) with MT1 and MT2 receptors antibodies. A) Immunoblot of rat hypothalamic tissue with anti-MT1 antibody. B) Same as in A but with anti-MT2 antibody.

Immunoblots of hypothalamic tissue indicated that labeling for MT1 and MT2 receptor antibodies was restricted to a single band of molecular weight greater than 37 kDa for MT1 and less than 37 kDa for MT2 (Fig. 1), within the reported molecular weights of both receptors between 28 and 40 kDa [2,28].

### 2.4. Immunofluorescence (IF)

IF was performed on free floating brain sections (70 µm) that were prepared as previously described. After rinsing several times with PBS, sections were incubated with block/permeablization solution (PBS containing 1% bovine serum albumin, 0.25% Triton-X100, and 5% normal goat serum) for 24 hours at 4ºC then rinsed with PBS for at least 10 minutes. Sections then were incubated with polyclonal MAP2 antibody (Abcam Inc., Cambridge, MA USA) at concentration of 1:100 overnight at 4ºC. Secondary antibody (Texas Red), (Abcam Inc.) was applied, after washing three times with PBS, and sections were incubated overnight at 4ºC. Negative controls were performed by omission of the primary antibody, which resulted in complete loss of signal. Sections were mounted as previously mentioned but the mounting media contained 1.5 mg/ml 4’, 6-diamidino-2-phenylindole, dilactate (DAPI) Images were obtained using a Zeiss LSM 510 META NLO confocal microscope with green HeNe 543 nm laser for Texas Red, and the Coherent Chameleon XR laser at 760 nm for DAPI in two-photon mode.

### 2.5. Image Analysis

Images were analyzed and quantified using Image J software (NIH). All images used for quantification purposes were obtained using identical illumination and exposure conditions. White balanced color images were converted to monochrome positive images using Adobe Photoshop ME 7.0. Pixel density was measured in a fixed area in the two SCNs per section (0.084 mm^2^) then averaged and normalized to an identical area outside the SCN. Normalized pixel densities in all sections (expressed as average ± standard error (SE)) were then averaged and compared at 4 different ZTs. As we used positive images for analysis the pixel density measured is now inversely proportional to the density of the label. Thus the lower the pixel density the darker is the image and therefore the higher receptor density and vice versa. Statistical analyses were performed using one-way ANOVA as well as Student’s T-test with Bonferoni adjustment. According to the Bonferoni adjustment the α level of significance or p value was calculated according to the following equation

\[ \alpha = {\rm{b/c}} \]

Where b is the probability of making one incorrect rejection, and c is the actual number of t-tests run. This procedure was performed to minimize the overall probability of making at least one incorrect rejection or ά which is derived from the following equation:

\[ \mathop \alpha \limits^\prime = 1 - {(1 - 0.05)^{\rm{c}}}{\rm{
                }}[13]. \]

## 3. Results

**Fig. 2:**
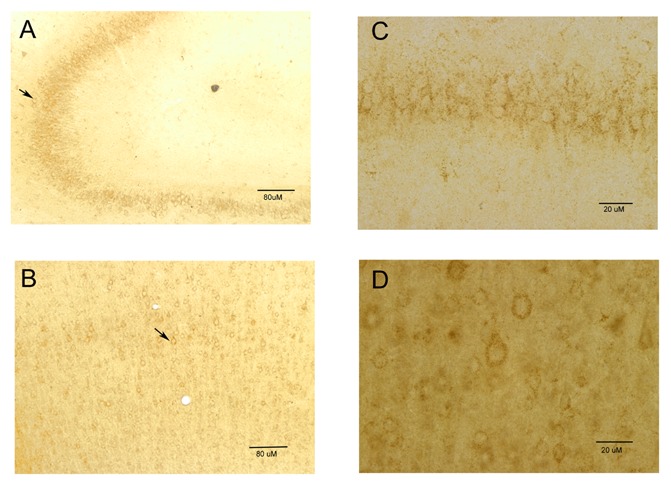
MT1 receptors localization in the cortex and the hippocampus. A) low and C) high magnification pictures showing label of hippocampal neurons with anti-MT1 antibody. Scale bars are 80 and 20 µm respectively. B) and D) show labeling with anti-MT1 antibody in cortical neurons in low and high magnification respectively. Scale bars are 80 and 20 µm respectively.

In the brain slices, labeling with MT1 (which consisted of a dark brown reaction product) was detected in the hippocampus and the cortex as positive controls. In the hippocampus labeling was found in CA1, CA2, and CA3 regions mainly around cell bodies (Fig. 2A and 2C). Label was also found in the cortex in the neurons of the deep layers and appeared to be restricted to the somas of the neurons and not the processes (Fig. 2B and 2D). We did not observe circadian changes in the pattern of MT1 receptor expression.

**Fig. 3:**
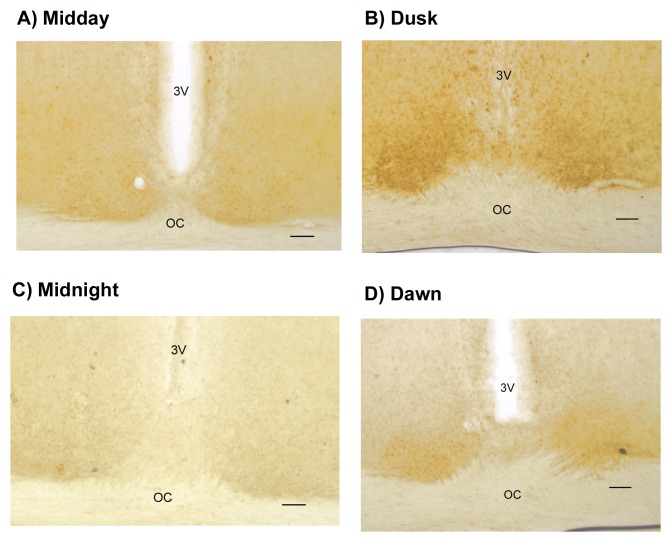
MT1 receptors localization in rat SCN across the circadian cycle. Labeling with MT1 antibody in the SCN is shown at A) midday ZT 5–7, B) dusk ZT 11–13, C) midnight ZT 23–01, and D) dawn ZT 17–19, respectively. 3V indicates the third ventricle, OC is the optic chiasm. Scale bar in each picture is 80 µm.

When the pattern of labeling for MT1 receptors within the SCN was examined across the circadian cycle, MT1 receptors were detected within the SCN particularly at dusk (ZT 11–13) (Fig. 3). Labeling was dense and had a diffuse pattern at that circadian time. In addition, labeling extended into the optic chiasm representing probably labeling of neuronal dendrites. There was no difference in distribution of the labeling within each SCN. During the other three circadian times: ZT 23–01 (dawn), ZT 5–7 (mid-day), and ZT 17–19 (midnight), labeling was also observed, but it was less pronounced when compared to the dusk period. Also the pattern of labeling was more discrete, probably labeling only somas of SCN neurons.

**Fig. 4:**
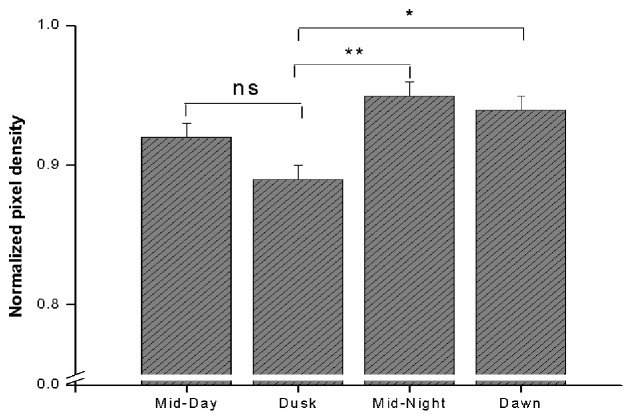
Circadian rhythm of MT1 receptors density in the SCN. Normalized pixel density is plotted at different CTs. * and ** indicates that p value is less than 0.5 and less than 0.01 respectively, ns statistically insignificant.

Our attempt of quantification confirmed that there was a significant variation in MT1 receptor density in the SCN across the circadian cycle, compared to non-SCN areas. One-way ANOVA indicated a significant daily variation in the density of label for MT1 in the SCN (p<0.001). Average MT1 receptor density in the SCN was significantly increased (indicated by low pixel values) at dusk compared to dawn (p < 0.05) and mid-night (p < 0.01) (Fig. 4). The average density of MT1 receptor label was higher at dusk than at midday; however this increase was statistically insignificant. On the other hand, MT1 receptor density compared to dawn and both midday and midnight was statistically insignificant, although the label was greater at dawn compared to these two circadian times.

**Fig. 5:**
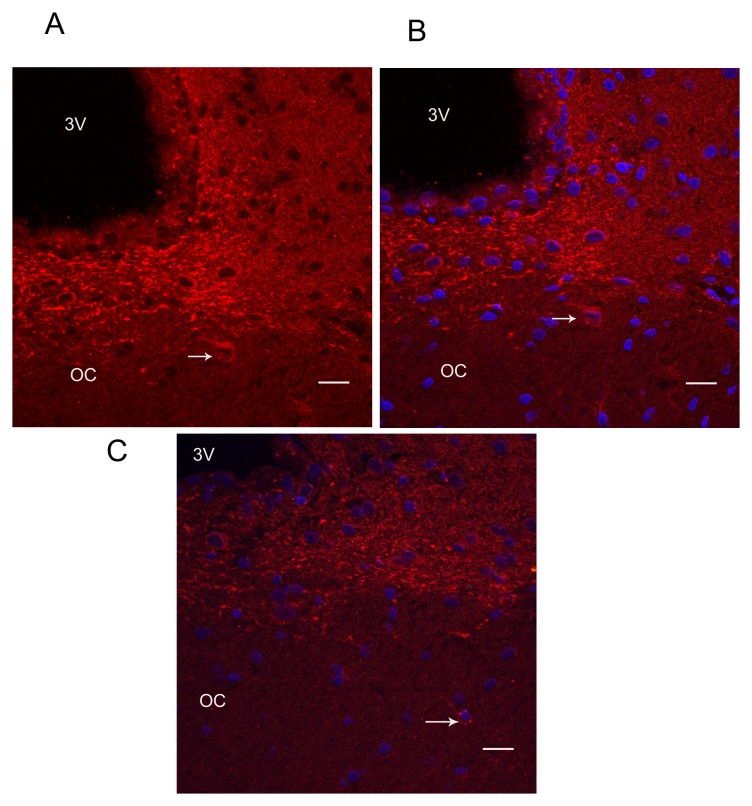
MAP2 staining in the SCN and optic chiasm. A) Labeling of the dendrites within the SCN is shown with red color. The arrow points to a neuron within the optic chiasm. B) An overlay of both DAPI labeling the nuclei (blue) and MAP2 labeling the dendrites (Red). C) Same as in B but in a different section; notice a more distant neuron within the SCN (arrows). 3V indicates the third ventricle. OC is the optic chiasm. Scale bar is 20 µm.

At midday (ZT 5–7) and dusk (ZT 11–13), label for MT1 extended into the optic chiasm apparently in dendritic processes. This identification was confirmed using antibody against MAP2 the dendrite marker [8]. This label probably originated from previously unobserved cell bodies in the optic chiasm that send dendritic processes into the SCN (Fig. 5).

**Fig. 6:**
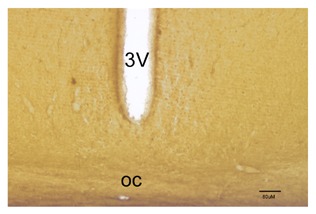
MT2 receptors immunostaining in the SCN. 3V indicates the third ventricle, OC is the optic chiasm. Scale bar is 80 µm.

**Fig. 7:**
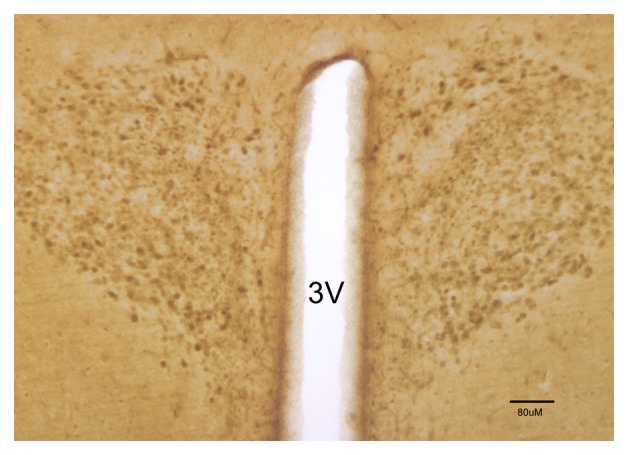
MT2 receptor immunolabeling in the paraventricular nucleus. 3V is the third ventricle. Scale bar is 80 µm.

No clear labeling for MT2 receptors was observed in the SCN at the same antibody concentration that was used for MT1 was detected at (1:500). When the concentration of the antibody was increased to 1:100, only minimal labeling was detected in the SCN (Fig. 6). On the other hand, at 1:100 concentration clear MT2 labeling was detected in the paraventricular nucleus (Fig. 7) as well as in the retina (data not shown).

## 4. Discussion

The results of this study show that the localization and distribution of melatonin receptors MT1 is regulated in a circadian mode in rat SCN. The highest observed receptor density was detected at dusk (ZT 11–13), which is the observed window of time for melatonin actions in SCN [5,9,12]. MT1 receptor density was elevated at dusk compared to that at dawn (ZT 23–01) and midnight (ZT17–19). Our attempt to quantify these changes showed that there was no statistically significant difference between MT1 receptor density at dusk and mid-day. These data suggest that the synthesis of melatonin receptors MT1 undergoes cyclic changes throughout the circadian cycle. Darkness signals conveyed to the SCN via the retinohypothalamic tract (RHT) result in MT1 receptor being synthesized and shuttled to the dendritic membrane, while during the day the receptors are degraded. This cyclic pattern of melatonin receptors expression and degradation fits perfectly with the observations that melatonin was most effective in influencing SCN function only at specific windows of time [5]. These data also agree with mRNA expression data that found a rhythm of expression similar to our model in rat SCN [15,18] as well as with data from binding studies [10]. Although Sugden et al. [26] did not find a statistically significant variation in MT1 mRNA receptor expression across the circadian cycle, they still found a pattern of variation in its expression similar to the model proposed here. However, and since this is a novel approach to define daily changes in melatonin receptor expression, further studies are needed to confirm these results using more advanced methods of quantification.

There were no apparent daily changes in MT1 labeling in the cortex and the hippocampus, but we did not test a number of rat brains large enough for statistical analysis to confirm this result.

The similar labeling pattern of MT1 and MAP2 suggests that MT1 receptor is mainly present in the dendrites and somas of SCN neurons. Moreover, labeling extended to what appears to be neurons within the optic chiasm (Fig. 3 and 5), which suggests the possibility of a population of SCN neurons within the optic chiasm that have a role in melatonin regulation of the circadian pacemaker. There is a possibility that our sectioning process displaced these neurons, but this possibility seems remote due to the consistency of our observation. However, further studies are needed to confirm this finding.

By contrast, MT2 receptors were not detected in this study. There is a possibility that the level of MT2 receptors in the SCN is not sufficient to be detected by this method at any time. This is more likely to be the case, as we were able to detect a very good signal with the MT2 antibody in the paraventricular nucleus (PVN) (Fig. 7) as well as in the western blots. This is also in agreement with mRNA data from Poirel et al. [18], who could not detect MT2 mRNA in SCN. However, we did not attempt to assess the daily pattern of changes in the PVN, which remains as a new venue to be explored.

The results suggest that melatonin action in the SCN could be attributed to MT1 receptors with a minimal role for MT2. This is in agreement with Weaver et al. [27], who found that MT2 receptors were not necessary to mediate either seasonal reproductive or circadian responses to melatonin. Meanwhile our results do not support other pharmacological studies that suggested that melatonin actions in the SCN were mediated via MT2 receptors [3,7]. However these studies relied on the use of two antagonists (luzindole and 4P-PDOT) that are not absolutely selective and may actually act as partial agonists for melatonin [2,16]. We have also found that these putative antagonists mimic some of melatonin electrophysiological effects in the SCN (29).

**Fig. 8:**
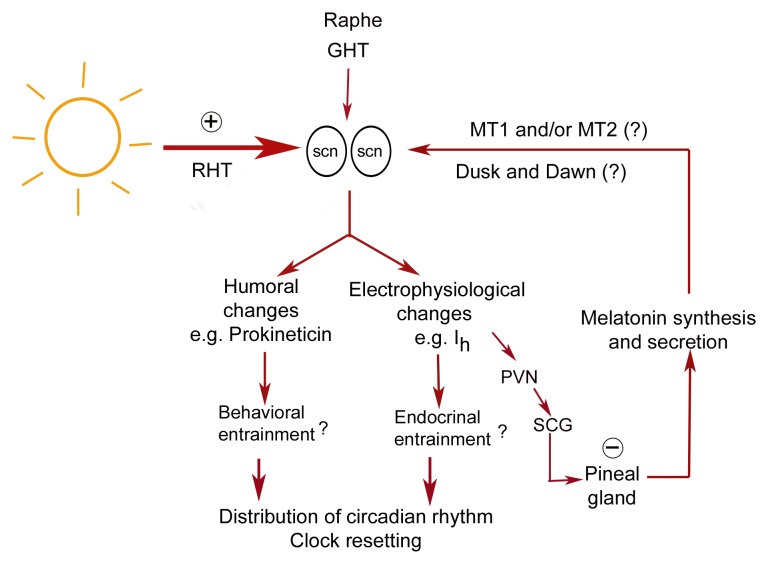
The possible feedback of melatonin on the SCN. RHT is the retinohypothalamic tract, GHT is the geniculohypothalamic tract, PVN is the paraventricular nucleus, and SCG is the superior cervical ganglia. Plus sign (+) indicates stimulation while negative sign (-) indicates inhibition.

In conclusion, melatonin receptor MT1 synthesis exhibited daily rhythmicity peaking at dusk and dawn, which would allow for melatonin to exert its action on the SCN at these specific windows of time. Figure 8 summarizes the possible input and output pathways of the SCN and the potential role of melatonin receptors. Further investigation is needed to confirm whether or not a functional MT2 receptor exists in the SCN and whether or not it exhibits circadian variation throughout the day.

This is the first demonstration of the daily pattern of melatonin receptor protein expression using the immunohistochemical approach. This is a step forward towards understanding of the role of melatonin in the regulation of the circadian clock, which can facilitate the therapeutic use of this hormone in jet lag and other sleep disorders.
